# Commentary: Contrasting motivational orientation and evaluative coding accounts: on the need to differentiate the effectors of approach/avoidance responses

**DOI:** 10.3389/fpsyg.2016.00163

**Published:** 2016-02-17

**Authors:** Andreas B. Eder, Klaus Rothermund, Bernhard Hommel

**Affiliations:** ^1^Psychology, University of WürzburgWürzburg, Germany; ^2^Department of Psychology, University of JenaJena, Germany; ^3^Department of Psychology, Leiden UniversityLeiden, Netherlands

**Keywords:** evaluative coding account, motivational systems, approach-avoidance, stimulus-response compatibility, Theory of Event Coding (TEC)

In a recent review paper on theoretical explanations of affective stimulus-response compatibility (aSRC) effects between positive/negative stimuli and approach/avoidance-related movements, Kozlik et al. (2015; KNL) argue that an evaluative-coding approach cannot fully account for aSRC effects with facial actions and that motivational orientations provide a better explanation. Their arguments are based on three observations that they consider incompatible with an evaluative-coding approach (Eder and Rothermund, 2008, ER) and the Theory of Event Coding (TEC; Hommel et al., 2001) from which the approach is derived: (1) attempts to dissociate evaluative coding and motivational orientation showed separable contributions from these two factors (Krieglmeyer et al., 2010); (2) aSRC can be easily changed in manual actions (by instructions of action labels) but hardly in facial actions (Neumann et al., 2014); and (3) the hemispheric asymmetry in the control of positive/negative facial expressions matches the hemispheric asymmetry assumed for motivational orientations (Davidson et al., 1990). In the following, we will explain why none of these three observations is inconsistent with an evaluative-coding approach and (4) why the motivational-systems approach represents little more than a rephrasing of observations. Rather than contrasting both accounts, we propose a theoretical integration of cognitive and motivational aspects as a more promising approach for future research.

Krieglmeyer et al. (2010) report a “motivational” compatibility effect (on top of an evaluative-coding effect) “that was independent of evaluative compatibility between stimulus valence and response label valence.” However, we note that this effect (a) actually interacted with evaluative compatibility (Exp. 1); (b) was markedly reduced (Exp. 2A) or eliminated (Exp. 2B) in non-evaluative tasks; and (c) is open to an alternative interpretation with internal recoding mechanisms. As noted by ER (Eder and Rothermund, 2008, Footnote 7), “internal recodings of action representations are likely …if perceptual action frames (e.g., moving the stimulus toward or away from the viewer) are more salient for action control.” Manikin movements toward and away from words may hence have triggered action re-coding in “toward” and “away” in some conditions, which explains a small instruction-independent aSRC effect.
2. We agree that smiling and frowning are linked to positive and negative affects, respectively, more rigidly than non-facial actions commonly are. We also agree that aSRC effects for facial responses are less affected by instructions of arbitrary action goals (e.g., performing “rain” and “sun” actions; Neumann et al., 2014). However, we fail to see why that might imply different mechanisms and explanations. In fact, the affectively extended TEC can easily account for all of these observations. According to TEC, perceptions and actions are represented by networks of distributed feature codes that represent their perceivable features. An action plan would thus code, among other things, the anticipated kinesthetic feedback from a smile or visual feedback after a lever movement. Considering that affect is derived from bodily sensations (James, 1884), feature networks should also incorporate codes representing the positivity/negativity of action consequences (Beckers et al., 2002; Eder et al., 2015). Perceptual and action events of the same valence then share feature codes—which explains aSCR (Eder and Klauer, 2007, 2009; Eder et al., 2012).
  Associations between movements and codes of their sensory consequences emerge through experience and as a function of contingency and contiguity, which also holds for facial expressions (Ekman et al., 1980; Kunde et al., 2011). The current action goal moderates feature networks by weighting features on goal-related dimensions more strongly (Memelink and Hommel, 2013), and verbal instructions can help to disambiguate goal-relevant action consequences (Eder et al., 2013). This means that the size of aSCR effects should depend on the strength of the acquired association and on the current goal/feature weighting (for evidence see Phaf et al., 2014). It makes sense to assume that affective consequences of smiling are more systematically/strongly associated with the responsible motor pattern than affective consequences of pulling something toward one's body. This implies that the relative contribution of previously acquired associations with affective codes is stronger for facial than for manual actions, which explains why the former are more immune to goal manipulations.
3. KNL cite several findings suggesting that facial expressions of happiness and of disgust are associated with particularly strong activation of left and right frontal cortex, respectively. While these observations actually do not speak to the involvement of motivational factors, KNL follow Davidson et al.'s (1990) terminology in simply labeling happiness an “approach emotion” and disgust a “withdrawal emotion.” Furthermore, previous research showed that only smiles that include activation of the *orbicularis pars lateralis* (Duchenne's marker) result in greater left frontal activation, while other smiles do not (Ekman and Davidson, 1993). Thus, even if we would agree to a close link between voluntary smiles and motivational brain systems, it is unclear whether Neumann et al.'s (2014) facial task without Duchenne's marker was sufficient to engage these systems.
4. Is KNL's “motivational orientation account” a reasonable theoretical alternative that explicates aSRC effects in terms of a different mechanism? Unfortunately, no mechanism is presented, which renders the referred-to “motivational orientations” theoretically empty boxes without additional explanatory value. “Explaining” approach-avoidance tendencies through activations of corresponding approach-avoidance systems is but a re-description of the to-be-explained phenomenon in allegedly explanatory terms, which we consider about as meaningful as, say, explaining perception through a “perception system.” Truly useful scientific models require the specification of the contents of motivation systems and the processes operating on these contents. How are positive and negative stimuli translated into corresponding behaviors? How does a “motivation system” select and energize movement patterns producing approach or avoidance? Our expanded TEC approach has the potential to provide answers to these critical questions, including those that KNL interpret as too challenging. Instead of contrasting both approaches, we suggest to integrate perceptual, emotional, and motivational aspects of human action into a unitary theoretical model that makes concrete, testable assumptions about the structures and processes of actions effecting approach and avoidance (see Eder and Hommel, 2013; Eder and Rothermund, 2013).

## Author contributions

All authors listed, have made substantial, direct and intellectual contribution to the work, and approved it for publication.

## Funding

Preparation of this comment was supported by grant ED 201/2-2 of the German Research Foundation (DFG) to AE.

### Conflict of interest statement

The authors declare that the research was conducted in the absence of any commercial or financial relationships that could be construed as a potential conflict of interest.
